# Patient with gastric cancer who underwent distal gastrectomy after treatment of COVID-19 infection diagnosed by preoperative PCR screening

**DOI:** 10.1186/s40792-022-01367-z

**Published:** 2022-01-17

**Authors:** Akiharu Kimura, Nobuhiro Morinaga, Wataru Wada, Kyoichi Ogata, Takayuki Okuyama, Hiroyuki Kato, Makoto Sohda, Ken Shirabe, Hiroshi Saeki

**Affiliations:** 1 Department of Surgery, Kiryu Kosei General Hospital, 6-3 Orihime-cho, Kiryu, Gunma 376-0024 Japan; 2 Department of General Surgical Science, Graduate School of Medicine, Gunma University, 3-39-22 Showa-machi, Maebashi, Gunma 371-8511 Japan

**Keywords:** COVID-19, Preoperative screening, Gastric cancer, Distal gastrectomy, Surgical smoke

## Abstract

**Background:**

Because of the coronavirus disease 2019 (COVID-19) pandemic, preoperative screenings for COVID-19 infection are often performed in many institutions. Some patients are diagnosed with COVID-19 infection by antigen tests or polymerase chain reaction (PCR) testing for COVID-19, even if they have no symptoms, such as fever or respiratory symptoms. We herein describe a patient with gastric cancer who underwent distal gastrectomy 6 weeks after recovering from COVID-19 infection diagnosed by preoperative PCR.

**Case presentation:**

An 86-year-old man was transferred to our hospital because of hematemesis and melena. A hemorrhagic gastric ulcer was found in the lesser curvature of the antrum by emergency endoscopy. Endoscopic hemostasis was performed, and he was discharged after recovery. A tumor-like lesion in the lesser curvature of the antrum was found on repeat endoscopy and was diagnosed as well-differentiated adenocarcinoma by biopsy. There was no evidence of lymph node metastasis or distant metastasis; therefore, we planned radical surgery. However, he was diagnosed with COVID-19 infection by preoperative PCR screening. Although he had no symptoms, such as fever or respiratory symptoms, he was hospitalized because of his advanced age. He was discharged 10 days after admission, and repeat COVID-19 PCR was negative. We planned radical surgery for the stomach tumor 6 weeks after recovery from the COVID-19 infection. A PCR-negative COVID-19 status was confirmed again before hospitalization. Open distal gastrectomy with Billroth I reconstruction was performed. We avoided ultrasonic scalpels and used a Crystal Vision 450D surgical smoke evacuator (I.C. Medical, Inc., Phoenix, AZ, USA) to reduce intraoperative surgical smoke. The postoperative course was uneventful.

**Conclusion:**

Because of the COVID-19 pandemic, some patients are diagnosed with COVID-19 infection by preoperative antigen tests or PCR, even if they have no symptoms. If possible, elective surgery should be performed 4 to 6 weeks after recovery from COVID-19 infection to maximize safety. Moreover, surgeons must consider intraoperative surgical smoke.

## Background

Because of the coronavirus disease 2019 (COVID-19) pandemic, some patients are diagnosed with COVID-19 infection by preoperative antigen tests or polymerase chain reaction (PCR) testing for COVID-19, even if they have no symptoms. Surgery in patients with perioperative COVID-19 infection increases the risk of respiratory complications and exposure for medical staff. If possible, elective surgery should be delayed for 4 to 6 weeks after a patient’s recovery [1]. We herein describe a case of gastric cancer in which we performed distal gastrectomy after the patient recovered from COVID-19 infection diagnosed by preoperative PCR screening.

## Case presentation

**Fig. 1:**
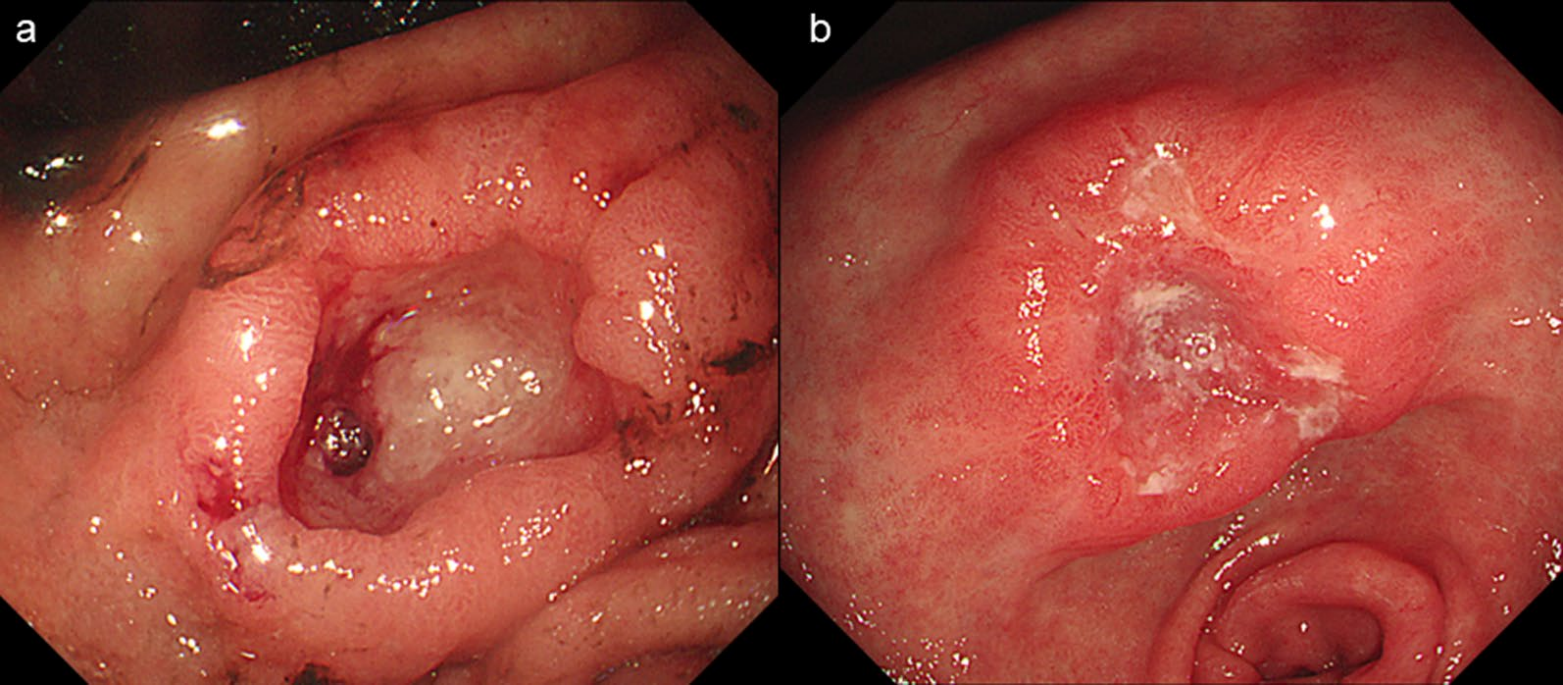
Endoscopy findings. **a** Emergency endoscopy at the first admission. A hemorrhagic gastric ulcer was found in the lesser curvature of the antrum. **b** Follow-up endoscopy showed a tumor-like lesion in the lesser curvature of the antrum that was diagnosed as well-differentiated adenocarcinoma by biopsy

**Fig. 2:**
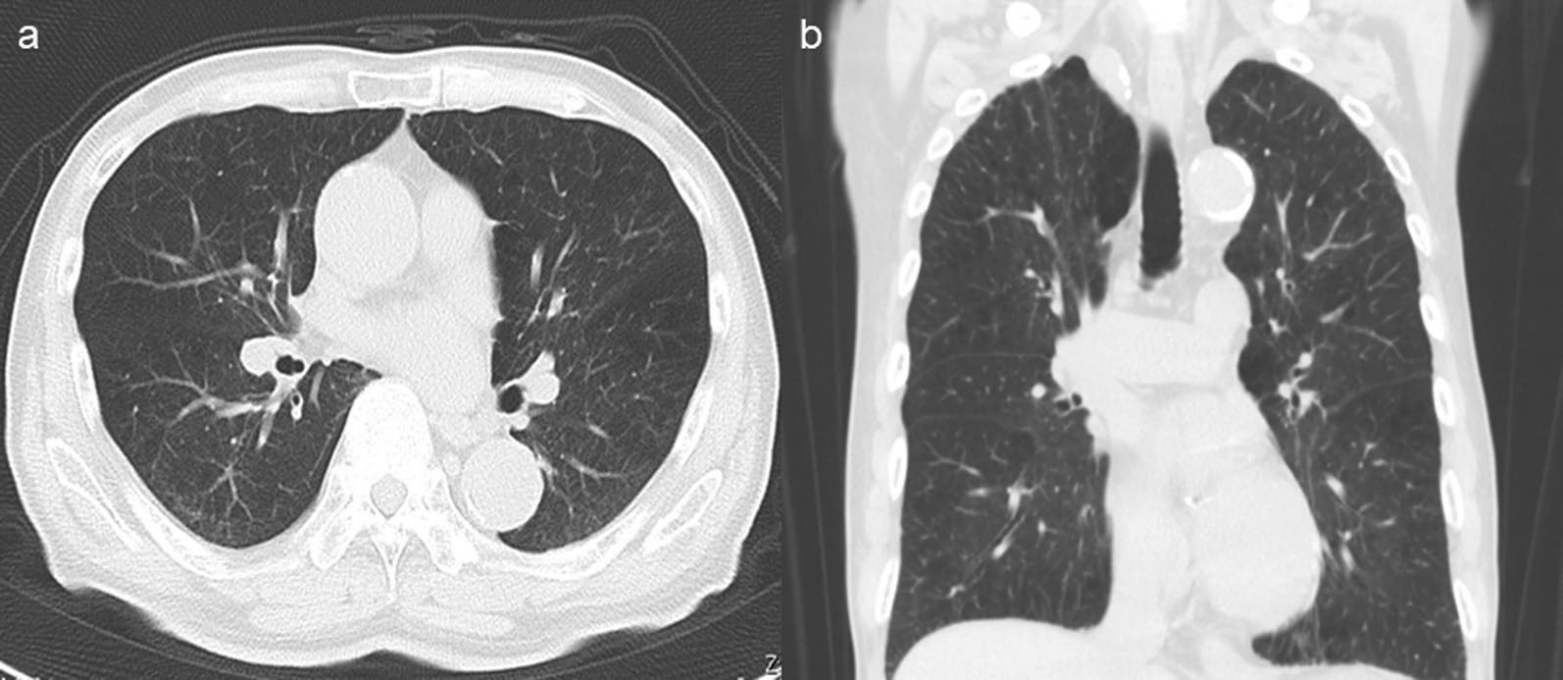
Chest computed tomography. There were no findings of pneumonia. **a** Axial view. **b** Coronal view

**Fig. 3:**
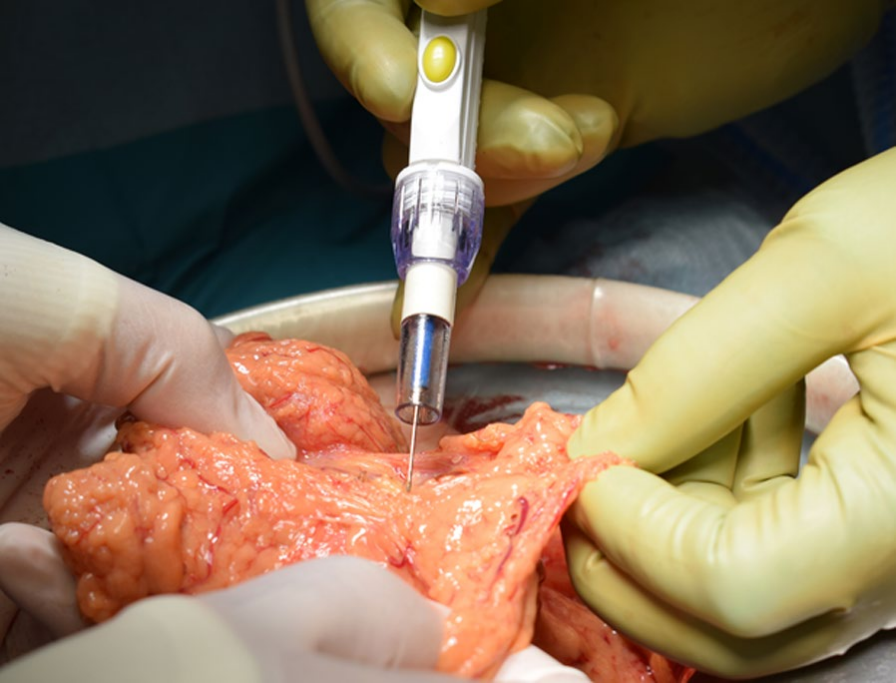
Operative findings. A Crystal Vision 450D surgical smoke evacuator was used to reduce surgical smoke during the operation

**Fig. 4:**
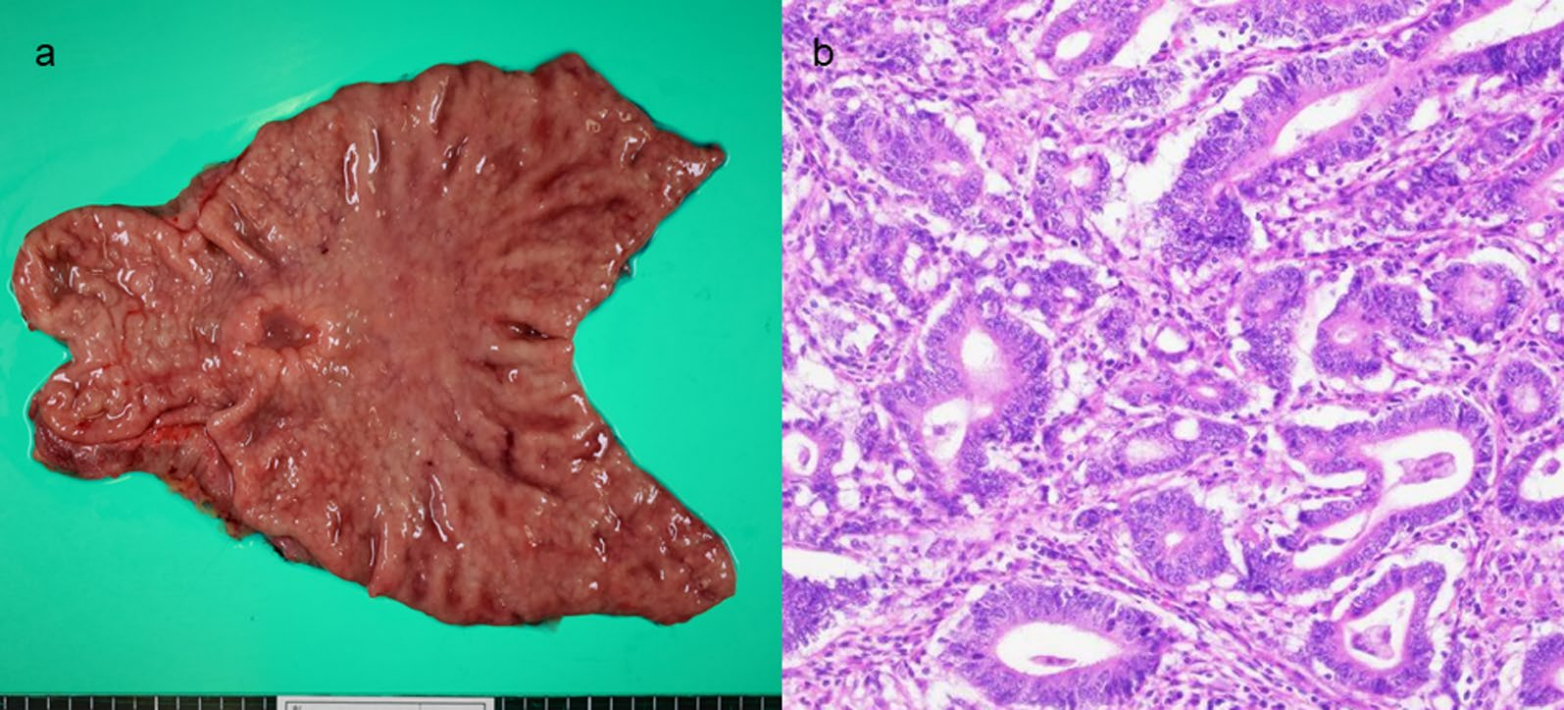
Resected specimen and pathological findings. **a** Resected specimen. Borrmann type 3 cancer was found in the lesser curvature of the antrum. **b** Pathological findings. Well- to moderately differentiated adenocarcinoma invading the subserosal layer (hematoxylin and eosin, × 400)

An 86-year-old man was transferred to our hospital because of hematemesis and melena. He had no obvious medical history other than hypertension. A hemorrhagic gastric ulcer was found in the lesser curvature of the antrum by emergency endoscopy (Fig. 1a). Endoscopic hemostasis with hypertonic saline–epinephrine and endoscopic hemoclips was performed. There was no evidence of bleeding after the procedure, and he was discharged 10 days after admission. Follow-up screening endoscopy was performed approximately 1 month after discharge. Endoscopy revealed a tumor-like lesion in the lesser curvature of the antrum that was diagnosed as well-differentiated adenocarcinoma by biopsy (Fig. 1b). Enhanced computed tomography (CT) revealed no evidence of lymph node metastasis or distant metastasis; therefore, we planned radical surgery. However, the patient was diagnosed with COVID-19 infection by preoperative PCR screening. He had no symptoms, such as fever or respiratory symptoms. CT did not identify pneumonia (Fig. 2). Because of his advanced age and the presence of cancer, he was at high risk of developing a serious clinical condition. As a result, he was hospitalized for follow-up observation. All members of the patient’s immediate family were negative for COVID-19 on PCR. He was discharged 10 days after admission with neither medication nor oxygenation because he met the criteria for discharge regarding COVID-19 infection, and we confirmed his PCR-negative status for COVID-19. At this time in our institution, we had no experience performing surgery under general anesthesia in patients with COVID-19 infection. Therefore, we consulted anesthetists and the infection control team to determine the appropriate timing of surgery after the patient’s recovery from COVID-19 infection. As a result, we planned radical surgery 6 weeks after he recovered from the infection. We repeated CT before surgery and found no evidence of progression of the gastric cancer or new development of pneumonia. We did not perform repeat endoscopy before surgery because of the exposure risk for COVID-19 infection. A PCR-negative status for COVID-19 was confirmed before hospitalization for surgery. At admission, the patient’s height was 162.7 cm, and he weighed 55.6 kg. Laboratory tests showed a white blood cell count of 6.0 × 10^9^/L, red blood cell count of 4.89 × 10^12^/L, and hemoglobin concentration of 15.3 g/dL Tumor markers, such as carbohydrate antigen 19-9 and carcinoembryonic antigen, were within the reference ranges. We initially planned laparoscopic gastrectomy for this patient. However, considering the COVID-19 infection, we decided to perform open gastrectomy to avoid the risk of surgical mist generated by laparoscopic surgery. Therefore, open distal gastrectomy with D1-plus lymph node dissection and Billroth I reconstruction was performed. The anesthetist and the operating room staff members used personal protective equipment and took measures to prevent infection during intubation. A muscle relaxant was used, and the cough reflex was avoided. We avoided ultrasonic scalpels and used a Crystal Vision 450D surgical smoke evacuator (I.C. Medical, Inc., Phoenix, AZ, USA) to reduce intraoperative surgical smoke (Fig. 3). The operation time was 165 min, and the blood loss volume was 80 mL. There was no evidence of respiratory complications perioperatively. The patient’s postoperative course was uneventful, and he was discharged from the hospital on the 18th day postoperatively. Microscopic examination of the gastric specimen revealed well- to moderately differentiated adenocarcinoma invading the subserosal layer without lymph node metastasis, described as pT3(SS)N0M0, pStageIIA in accordance with the TNM classification criteria (Fig. 4).

## Discussion

From 2 to 8 April 2020, a cross-sectional online survey of surgical practice was performed to evaluate screening policies for COVID-19 in surgical practice and to assess centers’ experiences with in-hospital COVID-19 infection [2]. A total of 936 centers in 71 countries responded to the survey. The study revealed that local guidelines in 71.9% of the centers recommended preoperative testing in the presence of symptoms or suspicious radiologic findings. Universal testing for every surgical patient was recommended in only 18.4% of the centers. Of the 936 centers, 295 centers experienced in-hospital COVID-19 infection, and 58 (19.7%) centers reported infections originating from asymptomatic patients or staff members. In the future, universal testing for every surgical patient should be warranted to clarify the prevalence of asymptomatic carriers, the potential impact on hospital outbreaks, and the cost–benefit balance of this testing.

In our institution, universal testing for every surgical patient, including those undergoing surgery with local anesthesia, has been performed since May 2020. In addition, testing for every inpatient has been performed before hospitalization since December 2020. The cost of testing for PCR is paid by public expenses. Initially, the turnaround time for PCR test results was 1 to 2 days. However, we can now perform PCR testing in our institution, and the results are available in 15 to 60 min. We performed 470 general surgeries in our institution from May 2020 to May 2021, and the current patient was the only patient diagnosed with COVID-19 infection by preoperative PCR screening.

A study published in 2020 revealed the postoperative outcomes of 1128 patients undergoing surgery who had COVID-19 infection confirmed within 7 days before or 30 days after surgery [3]. The study showed that the 30-day mortality rate was 23.8% (268/1128), and pulmonary complications occurred in 577 (51.2%) of the 1128 patients. In particular, 30-day mortality was associated with male sex, age of ≥ 70 years, malignant diagnosis, and emergency surgery. Lei et al. [4] analyzed the clinical data of 34 patients who underwent elective surgery during the incubation period of COVID-19. Fifteen (44.1%) patients required admission to the intensive care unit perioperatively, and 7 (20.5%) patients died after admission to the intensive care unit. Doglietto et al. [5] evaluated early surgical outcomes of patients with COVID-19 in Italy. They reported that the median chest radiography Brixia scores at admission and discharge were significantly higher in the correspondence of high complications.

Currently, most institutions screen for COVID-19 infection before surgery, and non-emergency surgery is postponed if patients have the infection. However, the appropriate timing of surgery after recovery from COVID-19 is controversial. Xiao et al. [6] reported that prolonged or intermittent viral shedding was found even after patients had recovered. Although the risk of contracting COVID-19 infection might be low in patients who have previously recovered from COVID-19, uncertainty remains regarding aerosolizing procedures, such as intubation [1]. Thyagarajan and Mondy [1] demonstrated that a symptom- or test-based strategy should be used to determine the timing of surgery after recovery from COVID-19. The authors recommended that surgical procedures should be delayed by 4 to 6 weeks, if possible. However, an international, multicenter, prospective cohort study by COVIDSurg Collaborative and GlobalSurg Collaborative showed that the risks of postoperative morbidity and mortality are greatest if patients undergo surgery within 6 weeks of diagnosis of COVID-19 infection [7]. They suggest that when possible, surgery should be delayed for at least 7 weeks following COVID-19 infection.

The patient described in the present report had advanced gastric cancer with bleeding at the initial visit. The risks of tumor progression and bleeding are of concern if an operation is delayed more than 7 weeks following COVID-19 infection. In the present case, we consulted with anesthetists and the infection control team regarding the appropriate timing of surgery after recovery from COVID-19 infection, and radical surgery was performed 6 weeks after recovery. As a result, there was no evidence of respiratory complications perioperatively, and the patient’s postoperative course was uneventful. Therefore, our plan for delayed surgery until after recovery from COVID-19 is considered appropriate.

The risks of surgical smoke generation in the COVID-19 pandemic were described by Chadi et al. [8]. The authors evaluated aerosols produced during dissection with electrosurgical instruments during both open and laparoscopic surgery. Many studies have shown that electrosurgical devices produce aerosolized bacteria and viruses, including human immunodeficiency virus, human papillomavirus, and hepatitis virus, which increase the risk of occupational exposure [9–12]. The use of such devices may increase the risk of infectious transmission of COVID-19. In particular, the risk of aerosol exposure for the surgical team may increase during laparoscopic surgery because of the artificial pneumoperitoneum [13]. Ultrasonic scalpels or electrical equipment, which are commonly used in laparoscopic surgery, may generate large amounts of surgical smoke. Li et al. [14] reported that after using electrical or ultrasonic equipment for 10 min, the particle concentration in the smoke during laparoscopic surgery was significantly higher than that during open surgery. Kameyama et al. [15] quantitated particulate matter counts as part of surgical smoke in 31 consecutive patients who underwent colectomy. However, they found that exposure to surgical smoke was lower during laparoscopic surgery than during open surgery for colorectal diseases. A systematic review by Matta et al. [16] showed no significant difference between smoke and aerosols generated from open surgery and those generated from minimally invasive surgery. We initially planned laparoscopic gastrectomy for our patient. However, after obtaining informed consent, we decided to perform open gastrectomy to avoid the risk of surgical mists generated by laparoscopic surgery. We avoided ultrasonic scalpels and used a surgical smoke evacuator (Crystal Vision 450D; I.C. Medical, Inc.) to reduce intraoperative surgical smoke as much as possible. Ekci [17] devised an easy-to-use electrocautery smoke evacuation device for open surgery for use with existing suction tubes and electrocautery devices. The authors demonstrated that this surgical smoke evacuation device provided a convenient and effective method of smoke evacuation during open surgery. Tokuda et al. [18] used a ConMed Aer Defence (Japan Medicalnext Co., Ltd., Tokyo, Japan) as the surgical smoke evacuation system. The authors demonstrated that the average total volatile organic compound concentration in the operating room was significantly lower when the evacuation system was used. In our case, there was no evidence of respiratory complications perioperatively because of appropriate management after COVID-19 infection and careful procedures during the operation. In the future, after evidence for operative cases following COVID-19 infection has been accumulated and when protective measures can be implemented, laparoscopic surgery might be performed instead of open surgery for less invasive procedures. Moreover, vessel-sealing systems instead of ultrasonic scalpels might be useful to reduce surgical mist during laparoscopic surgery.

## Conclusion

We have herein described a case of gastric cancer for which we performed distal gastrectomy 6 weeks after the patient recovered from COVID-19 infection diagnosed by preoperative PCR screening. Because of the COVID-19 pandemic, some patients are diagnosed with COVID-19 infection by preoperative screening using antigen tests or PCR tests, even if they have no symptoms. If possible, elective surgery should be delayed for 4 to 6 weeks after recovery from COVID-19 to maximize safety and prevent respiratory complications. Moreover, surgeons must consider surgical smoke during the operation.

## Data Availability

All data generated or analyzed during this study are included in the published article.

## Abbreviations

COVID-19: Coronavirus disease 2019
CT: Computed tomography
PCR: Polymerase chain reaction
